# Circular RNA-based liquid biopsy: a promising approach for monitoring drug resistance in cancer

**DOI:** 10.20517/cdr.2025.142

**Published:** 2025-10-30

**Authors:** Desh Deepak Singh, Dharmendra Kumar Yadav, Dongyun Shin

**Affiliations:** ^1^Amity Institute of Biotechnology, Amity University Rajasthan, Jaipur 303002, India.; ^2^College of Pharmacy, Gachon University, Incheon 21924, Republic of Korea.

**Keywords:** Liquid biopsy, circular RNAs (circRNAs), drug resistance, cancer biomarkers, non-invasive monitoring

## Abstract

Drug resistance remains a significant challenge in achieving successful cancer treatment, often leading to disease recurrence and reduced patient survival. While traditional tissue biopsies provide valuable insights into tumor biology, they are invasive, infrequent, and may fail to capture the full complexity of tumor heterogeneity and dynamic molecular changes. In contrast, liquid biopsy has emerged as a minimally invasive, real-time approach for monitoring tumor evolution through the analysis of circulating biomarkers. Among these biomarkers, circular RNAs (circRNAs) - a distinct class of non-coding RNAs characterized by covalently closed-loop structures - have gained attention due to their remarkable stability, abundance in body fluids, and functional involvement in gene regulation. Increasing evidence supports the role of circRNAs in mediating drug resistance through mechanisms such as inhibition of apoptosis, epithelial-mesenchymal transition, autophagy, and drug efflux, largely via interactions with microRNAs or proteins. Advanced detection methods, including quantitative reverse transcription polymerase chain reaction, droplet digital polymerase chain reaction, and RNA sequencing, combined with computational tools, enable precise profiling of circRNAs in plasma or exosomes. CircRNA-based liquid biopsies offer a dynamic, non-invasive strategy for early detection of therapeutic resistance and may guide personalized treatment decisions. This review highlights the technological advancements, biological relevance, and clinical promise of circRNAs as circulating biomarkers, emphasizing their potential in precision oncology and future collaborative translational applications.

## INTRODUCTION

The emergence of drug resistance in cancer therapy is one of the greatest obstacles to long-term therapeutic success. Even with advances in chemotherapy, targeted therapies, and immunotherapies, many solid tumors relapse in patients due to either intrinsic or acquired resistance mechanisms^[1]^. While biopsies are considered the gold standard for tumor characterization, tissue sampling is invasive, usually performed only once at diagnosis, and fails to capture the temporal and spatial heterogeneity of tumor biology^[2]^. As precision oncology advances, there is a need for dynamic and non-invasive methods to assess cancer progression and therapy response^[3]^. Liquid biopsy presents this opportunity by assessing tumor-derived elements [circulating tumor cells (CTCs), circulating tumor DNA (ctDNA), *etc.*] from biofluids, most commonly blood, urine, or saliva^[4]^. New markers in liquid biopsy include circular RNAs (circRNAs) and other non-coding RNAs, which have garnered attention as potential biomarkers in the context of drug resistance due to their structural stability, abundance, linearity of representation, and biologically relevant functional states^[5]^. CircRNAs represent a class of endogenous non-coding RNAs identified by a covalently closed-loop structure that is resistant to degradation by exonucleases^[6]^. Their circular structure confers exceptional stability in bodily fluids, making them promising candidates for biomarker discovery^[7]^. CircRNAs were initially dismissed as transcriptional noise, but they are now recognized as regulators of biological processes such as proliferation, apoptosis, autophagy, and gene expression^[8]^. A growing body of evidence has shown that circRNAs are involved in the development of resistance to anticancer therapies through multiple mechanisms, including sponging microRNAs (miRNAs), interacting with RNA-binding proteins (RBP), regulating signal transduction pathways, and modulating transcription^[9]^.

The finding of circulating circRNAs through liquid biopsy represents a new frontier for non-invasive monitoring of drug resistance in a cancer context^[10]^. Given that circRNAs are stable in plasma and other fluids, we can readily quantify circRNA presence and abundance through innovative molecular techniques, including quantitative reverse transcription polymerase chain reaction (qRT-PCR), RNA sequencing (RNA-seq), and droplet digital PCR (ddPCR)^[5]^. Therefore, researchers have been able to link resistance to specific therapies with cancer-specific circRNA signatures^[11]^. For example, in non-small cell lung cancer (NSCLC), circRNA_102231 was shown to be overexpressed in cases where NSCLC patients had resistance to gefitinib, an epidermal growth factor receptor (EGFR)-tyrosine kinase inhibitor (TKI)^[12]^. The mechanism involved acting as a sponge to miR-130a-3p, which resulted in upregulation of oncogenic miR targets. As another example, in the context of breast cancer, circRNA cerebellar degeneration-related protein 1 gene (CDR1) antisense RNA (CDR1as) was correlated with tamoxifen resistance through modulation of the miR-7/EGFR pathway^[13]^. Cancer drug resistance is often multifaceted and involves changes in drug metabolism, expression of efflux pumps, DNA repair, epithelial-mesenchymal transition (EMT), and stemness. CircRNAs have the potential to disrupt many of these pathways^[14]^. For example, circHIPK3 has been shown to support chemoresistance by targeting various downstream processes in colorectal and bladder cancers^[15]^. The involvement of circRNAs in modulating resistance at multiple levels highlights their promise as unifying biomarkers that profile the changing resistance landscape during treatment^[16]^. In addition, it is plausible that circRNAs may provide a better biological picture of tumor heterogeneity than a biopsy from only one tumor site, given that circRNAs may be from diverse tumor sites^[17]^. Despite the very optimistic possibilities, challenges remain in circulating circRNAs for clinical purposes^[18]^. First, protocols for sample collection, RNA isolation, and data normalization must be established to ascertain reproducibility. Second, there may be detection and validation issues with lowly abundant circRNAs^[19]^; circumvention of low amounts of rare circRNAs may require a very sensitive detection method^[20]^. A third significant challenge is to reliably differentiate tumor-derived circRNAs from circRNAs in normal tissues^[21]^. Fourth, circRNAs with significant clinical value must be further validated in extensive, multi-center translational studies and matched to numerous cancer types and treatment approaches^[22]^. The detection of circulating circRNAs using liquid biopsy represents a watershed innovation in cancer diagnostics and therapeutic monitoring^[22]^. Real-time detection of molecular underpinnings of drug resistance would enhance personalized treatment through circRNA profiling to guide drug choice and ultimately improve patient outcomes^[23]^. As this exciting field develops further, circRNA biomarkers are expected to be integrated into clinical practice, facilitating more adaptive cancer care within the context of personalized medicine.

## CIRCRNAS FOR TRACKING DRUG RESISTANCE IN CANCER

**Figure 1:**
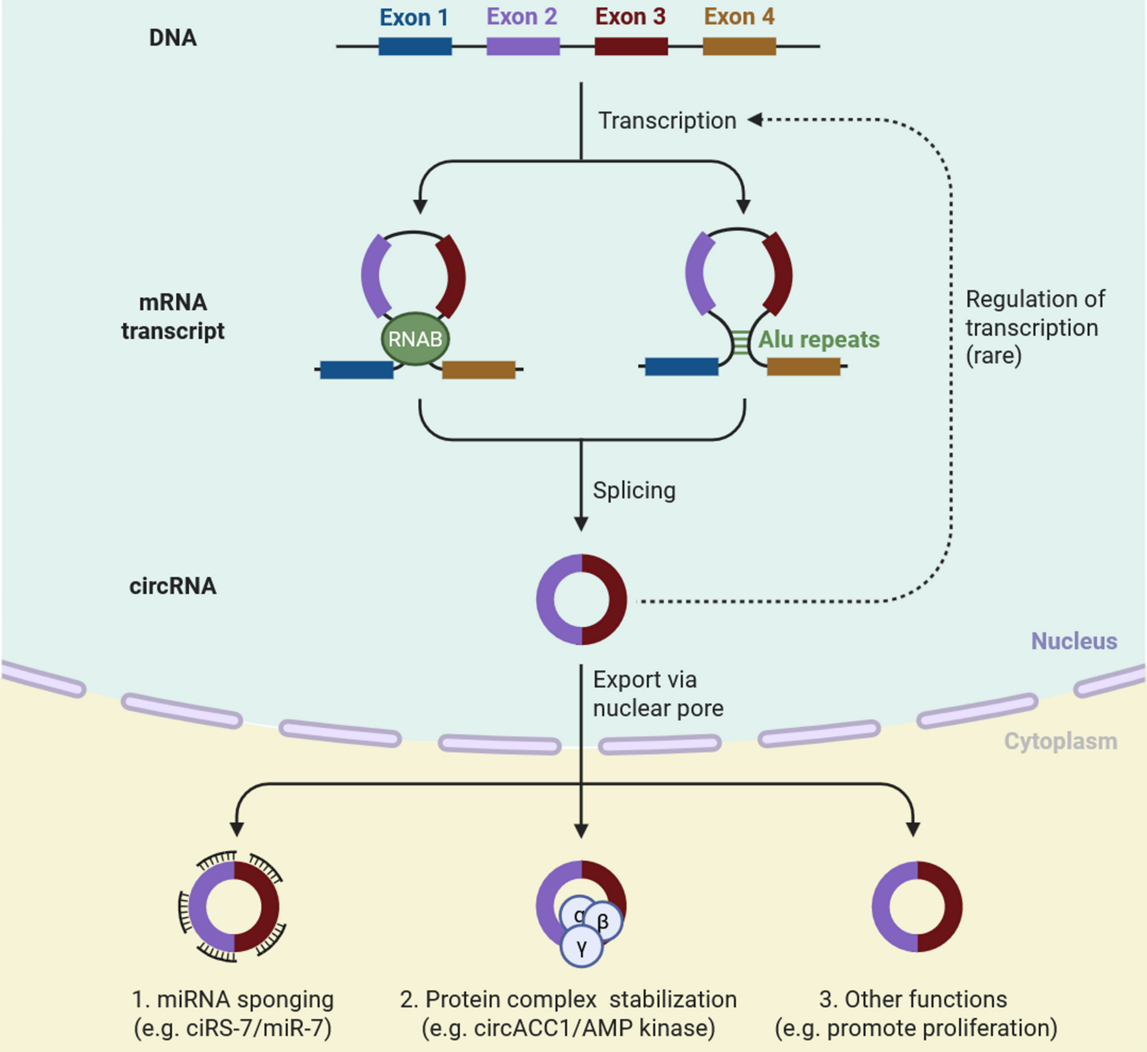
This schematic illustrates the formation of circRNAs through back-splicing of pre-mRNA, where exons are circularized via RBP or Alu repeat elements. The resulting circRNA is exported from the nucleus into the cytoplasm. CircRNAs can perform multiple regulatory functions, including (1) miRNA sponging (e.g., ciRS-7 sequestering miR-7); (2) stabilization of protein complexes (e.g., circACC1 supporting AMP kinase activity); and (3) other cellular roles, such as promoting proliferation and modulating gene expression. Though rare, circRNAs may also influence transcriptional regulation in the nucleus. These diverse functions contribute to circRNAs’ roles in cancer progression and drug resistance [Created in BioRender. Singh DD (2025)]. circRNAs: Circular RNAs; RBP: RNA-binding proteins; miRNA: microRNA; AMP: adenosine monophosphate.

CircRNAs are generated from pre-mRNA transcripts through a unique phenomenon known as back-splicing. Back-splicing is an event where a downstream splice donor connects to an upstream splice acceptor^[24]^. Back-splicing can occur with the help and support of RBP or inverted repeat elements - sequences that bring exons into close proximity, enabling their circularization^[25]^. The resulting circRNAs consist of covalently closed loop structures and therefore lack 5′ caps or 3′ poly (A) tails^[26]^. The absence of these terminal modifications contributes to the very high stability of circRNAs compared with linear RNAs [Figure 1]^[27]^. CircRNAs can additionally stabilize protein complexes, as demonstrated by circACC1, which aids in the assembly of the adenosine monophosphate (AMP) kinase complex and has a role in metabolism^[28]^. CircRNAs have also been associated with promoting proliferation, survival, and resistance to therapy in cancer cells by interacting with major signal transduction pathways^[29]^. They are not commonly involved in the regulation of transcription in the nucleus; however, there is an emerging role (either positive or negative) for some circRNAs to direct gene expression in multiple ways [Figure 1]^[30]^. Once formed, circRNAs will be exported from the nucleus to the cytoplasm via nuclear pores^[31]^. Once in the cytoplasm, they will exert many biological functions. One of the most reported is miRNA sponging^[32]^. CircRNAs contain multiple binding sites for specific miRNAs. By binding these miRNAs, circRNAs can sequester them away from their target mRNAs and all their splicing isoforms, thereby inhibiting their regulatory effects^[32]^. For example, ciRS-7 acts specifically as a sponge for the miR-7 pathway, affecting oncogenic pathways. Studies have established certain circRNAs, such as circHIPK3, circFOXO3, and circRNA_100290, which can modulate some cancer pathways and affect chemotherapy sensitivity^[32]^. CircHIPK3 is one of the well-studied circRNAs and has been reported to be overexpressed in colorectal, lung, and bladder cancer^[33]^. This circRNA functions as a sponge for multiple tumor-suppressive miRNAs, such as miR-124 and miR-558, and promotes cell proliferation and chemoresistance to 5-fluorouracil (5-FU) and cisplatin^[34]^. Another significant circRNA is circRNA forkhead box O3 (circFOXO3), which has been shown to bind to cyclin-dependent kinase 2 (CDK2) and p21, and affect cell cycle regulation and apoptosis in breast and lung cancer. Dysregulation of circFOXO3 has also been implicated with chemoresistance and poor clinical outcomes^[35]^. CircRNA plasmacytoma variant translocation 1 (Circ-PVT1) participates in paclitaxel resistance in gastric cancer as a sponge for miR-124-3p and regulates the EMT marker zinc finger E-box-binding homeobox 1 (ZEB1), thus promoting invasive and drug-resistant phenotypes; circ-MTO1 in hepatocellular carcinoma (HCC) promotes doxorubicin sensitivity by sponging miR-9 and upregulating the tumor suppressor p21, and its downregulation correlates with increased resistance and poorer prognosis^[36]^. Circ-AKT3 regulates temozolomide (TMZ) resistance in glioblastoma (GBM) by regulating the phosphoinositide 3-kinase (PI3K)/protein kinase B (AKT) pathways and cancer stem cell characteristics^[37]^. Circ-ABCB10, another extensively studied circRNA, provides resistance to multiple drugs in lung and breast cancer by regulating B-cell lymphoma 2 (BCL2) through the modulation of miR-1271^[18]^. As circRNAs continue to advance, future endeavors should address resistance issues, such as integrating circRNA panels into monitoring workflows, and further evaluating and validating circRNAs as therapeutic targets to reverse drug resistance and improve patient outcomes^[9]^. Key circRNAs implicated in cancer drug resistance, along with their pathways, mechanisms, and clinical applications, are presented in Table 1.

**Table 1:** Key circRNAs implicated in cancer drug resistance: pathways, mechanisms, and clinical applications

**S.N.**	**CircRNA name**	**Tumor type**	**Target pathway/gene**	**Mechanism of drug resistance**	**Clinical relevance/potential use**	**Ref.**
1	circHIPK3	Colorectal, lung, bladder	miR-124, miR-558	Acts as a sponge to suppress tumor-suppressor miRNAs; promotes resistance to 5-FU and cisplatin	Biomarker for chemotherapy resistance	[33]
2	circFOXO3	Breast, lung, gastric	FOXO3, p21, CDK2	Interferes with cell cycle regulation and apoptosis pathways	Prognostic marker; potential therapeutic target	[38]
3	circRNA_100290	Oral squamous cell carcinoma	miR-29 family	Modulates cell proliferation and cisplatin resistance	Diagnostic and drug response predictor	[39]
4	circ_0001946	NSCLC	miR-135a-5p, STAT6	Promotes gefitinib resistance by activating STAT6/PI3K/AKT pathway	Potential marker for EGFR-TKI resistance monitoring	[40]
5	circRNA CDR1as	Glioma, breast	miR-7, EGFR pathway	Regulates drug response via miRNA sponging and EGFR signaling	Associated with resistance to targeted therapy	[41]
6	circ-PVT1	Gastric cancer	miR-124-3p, ZEB1	Facilitates paclitaxel resistance by modulating EMT	Marker of chemoresistance and poor prognosis	[36]
7	circMTO1	HCC	miR-9/p21	Enhances doxorubicin sensitivity via tumor suppressor pathways	Therapeutic sensitization target	[42]
8	circAKT3	GBM	PI3K/AKT pathway	Promotes TMZ resistance via maintaining stemness	Candidate for targeting glioma stem cells	[43]
9	circ-ABCB10	Breast, lung cancer	miR-1271, BCL2	Enhances resistance by modulating apoptosis and cell survival	Liquid biopsy candidate for resistance monitoring	[29]

circRNAs: Circular RNAs; miRNAs: microRNAs; 5-FU: 5-fluorouracil; FOXO3: forkhead box O3; CDK2: cyclin-dependent kinase 2; NSCLC: non-small cell lung cancer; STAT6: signal transducer and activator of transcription 6; PI3K: phosphoinositide 3-kinase; AKT: protein kinase B; EGFR-TKI: epidermal growth factor receptor-tyrosine kinase inhibitor; ZEB1: zinc finger E-box-binding homeobox 1; EMT: epithelial-mesenchymal transition; HCC: hepatocellular carcinoma; GBM: glioblastoma; TMZ: temozolomide; BCL2: B-cell lymphoma 2.

## LIQUID BIOPSY-BASED DETECTION OF CIRCULATING CIRCRNAS

Cancer treatment has evolved considerably with the introduction of targeted therapies, immunotherapies, and combination regimens^[29]^. Unfortunately, drug resistance (whether intrinsic or acquired) remains a significant challenge that can culminate in therapeutic failure, progression of disease, and suboptimal survival^[44]^. Due to the dynamic and heterogeneous nature of tumors, it is wise to continually assess molecular changes during treatment^[6]^. Although tissue biopsies can be educational, they are invasive, typically only provide a limited evaluation of tumor biology, and cannot be used for longitudinal monitoring^[45]^. As a result, liquid biopsy has emerged as a novel, non-invasive application to assess real-time cancer progression and treatment resistance^[46]^. Table 2 presents a comparative analysis of the components involved in liquid biopsy-based detection of circulating circRNAs for monitoring drug resistance in cancer^[55]^. Liquid biopsy is the characterization of tumor-derived constituents, such as CTCs, ctDNA, extracellular vesicles, and different species of RNA, including circRNAs, in biofluids (e.g., blood)^[56]^. Due to their closed-loop structure that lacks free 5′ and 3′ ends, circRNAs resist exonuclease degradation, enabling them to freely circulate and serve as stable and strong biomarkers^[6]^. In the cancer context, circRNAs can exhibit multiple roles, e.g., serving as a miRNA sponge, interacting with RBP, regulating transcription, and even translated into functional peptides^[16,57]^. Importantly, dysregulated circRNA expression could result in mechanisms of drug resistance across multiple malignancies, such as lung, breast, liver, and colon cancer^[44]^. CircRNAs detected through liquid biopsy will have a multitude of advantages for the clinic, many based on what we discussed in the previous sections^[24]^. First, circRNAs in liquid biopsies are exceptionally stable, allowing their detection in biofluids often without immediate specimen processing^[58]^. Second, liquid biopsies enable clinicians to collect real-time samples from patients multiple times, providing insights into system or tumor responses and facilitating early detection of resistance^[2]^. Third, liquid biopsies are non-invasive and can obtain biospecimens from patients who might otherwise be ineligible due to poor general condition, prior trauma, or advanced disease^[59]^. Fourth, circulating circRNAs can originate from multiple tumor sites, offering a more comprehensive view of the disease and potentially revealing emerging resistance profiles simultaneously^[59,60]^. Recent advancements in technology have enabled sensitive and specific detection of circRNAs from plasma and serum samples using qRT-PCR, ddPCR and RNA-seq methods^[60]^. These platforms allow for profiling circRNA expression changes during treatment, which may provide early signals of therapeutic failure or developing resistance. Numerous preclinical and clinical studies have identified circRNA signatures correlated with chemoresistance, resistance to tyrosine kinase inhibitors, and resistance to immune checkpoint blockade, highlighting their translational potential^[61]^. Despite these promising advancements, challenges exist. There remains a lack of standardization for the many pre-analysis variables that can influence circRNA measurements (e.g., sample processing in clinical laboratories, RNA isolation protocols, and data normalization)^[62]^. Additionally, further validation through large, multi-center clinical studies is required to assess the diagnostic or prognostic value of specific circRNAs. Combining circRNA biomarkers with other molecular and clinical measures may enhance their predictive power and increase their utility in personalized medicine^[63]^. In summary, the liquid biopsy-based detection of circulating circRNAs provides a new, effective paradigm for non-invasive tracking of drug resistance in cancer^[6]^. As research continues and the technology advances, it is possible that circRNA profiling will become a relevant component of the clinical oncology arsenal, enabling real-time treatment guidance for drug resistance and improving outcomes in the era of precision oncology^[44]^.

**Table 2:** Components of liquid biopsy-based detection of circulating circRNAs for drug resistance monitoring in cancer: a comparative analysis

**S.N.**	**Component category**	**Specific component**	**Description/principle**	**Advantages**	**Limitations/disadvantages**	**Ref.**
1	Biological sample	Plasma/serum	Primary source of circulating circRNAs; obtained from peripheral blood	Minimally invasive; widely used; good RNA stability	Low RNA yield; variability depending on handling	[5]
2	Biological sample	Urine/saliva/CSF	Alternative fluids for site-specific cancers (e.g., urological, CNS)	Non-invasive; can reflect local tumor biology	Lower circRNA concentrations; limited standard protocols	[6]
3	Cellular/molecular assay	Exosome isolation	Isolation of tumor-derived exosomes containing circRNAs	Enhances tumor specificity; protects RNA from degradation	Time-consuming; requires specialized kits or equipment	[47]
4	Cellular/molecular assay	RNase R treatment	Digests linear RNAs to enrich circRNAs	Increases specificity for circRNAs	May result in partial RNA loss if not optimized	[35]
5	RNA extraction method	Column-based/magnetic bead kits	Commercial kits (e.g., Qiagen, Norgen) used for isolating total RNA from biofluids	High RNA purity; optimized for low-input samples	Costly; requires careful sample handling	[48]
6	Detection principle	qRT-PCR with divergent primers	Amplifies the back-splice junction unique to circRNAs	Cost-effective; highly specific for known circRNAs	Limited to known targets; less sensitive for low-abundance circRNAs	[49]
7	Detection principle	ddPCR	Uses microdroplets to detect and quantify RNA copies with high sensitivity	Absolute quantification; ideal for rare circRNAs	Expensive; requires specialized instruments	[50]
8	Detection principle	RNA-seq	Unbiased sequencing to detect both known and novel circRNAs	Comprehensive; detects novel circRNAs and expression changes	Data-intensive; needs bioinformatics expertise	[51]
9	Bioinformatics analysis	CIRCexplorer2, find_circ, CIRI2, DCC	Tools used to identify circRNAs from sequencing data by mapping back-splice junctions	Enables genome-wide circRNA discovery	Complex pipelines; prone to false positives without proper filtering	[52]
10	Bioinformatics analysis	circBase, CircInteractome, circAtlas	Databases for annotation, interaction prediction, and biological interpretation of circRNAs	Provides functional context and regulatory network insights	Limited clinical annotations for novel circRNAs	[53]
11	Clinical relevance	Resistance monitoring	Real-time, longitudinal assessment of treatment response via circRNA dynamics	Guides therapy decisions; non-invasive surveillance	Not yet standardized for clinical use; requires large-scale validation	[45]
12	Clinical relevance	Personalized treatment adaptation	Using circRNA expression changes to tailor treatment regimens dynamically	Supports precision oncology	Translational challenges; regulatory hurdles	[54]

circRNAs: Circular RNAs; CSF: cerebrospinal fluid; CNS: central nervous system; qRT-PCR: quantitative reverse transcription polymerase chain reaction; ddPCR: droplet digital polymerase chain reaction; RNA-seq: RNA sequencing; DCC: deleted in colorectal carcinoma.

## CIRCULATING CIRCRNAS AS EMERGING BIOMARKERS IN LIQUID BIOPSY FOR DRUG RESISTANCE SURVEILLANCE IN CANCER

**Figure 2:**
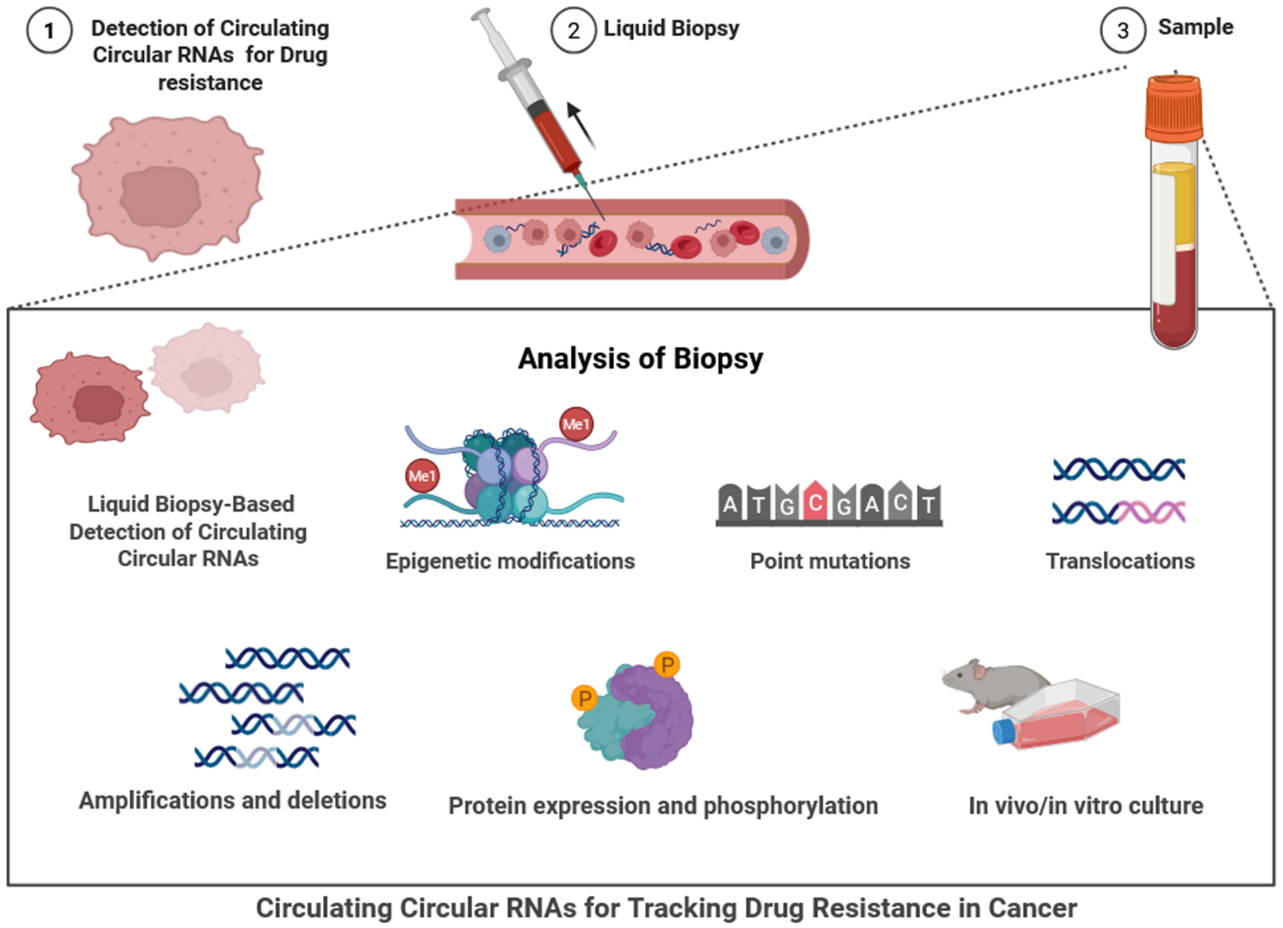
The figure depicts the use of liquid biopsy-based detection of circulating circRNAs to track drug resistance in cancer, beginning with the identification of drug-resistant tumor cells (Step 1) and progressing to liquid biopsy collection via blood draw and non-invasive material (Step 2). The resulting sample (Step 3), generally plasma or serum, will undergo analysis for molecular changes associated with the resistance. The analysis options include various molecular methods to detect epigenetic modifications, point mutations, translocations, and copy number changes such as amplifications or deletions. Other assessments also include protein expression and phosphorylated proteins, as well as the use of *in vivo*/*in vitro* methodologies to establish functional changes. Importantly, the aim is to detect and characterize the stable, circulating circRNAs in the liquid biopsy, where they may serve as potential circulating biomarkers. The proposed workflow enables real-time, non-invasive capture of valuable information regarding therapeutic resistance and supports the implementation of precision oncology and therapies for cancer treatment [Created in BioRender. Singh DD (2025)]. circRNAs: Circular RNAs.

CircRNAs are becoming increasingly popular as circulating biomarkers. Evidence of their dysregulation in numerous cancers adds to our knowledge of resistance mechanisms and a bright future for therapeutic monitoring^[15]^. The liquid biopsy-based approach for detecting circulating circRNAs is used to monitor drug resistance in cancer^[15]^. The process begins with the identification of resistant cancer cells and the collection of a blood sample, from which plasma or serum is isolated^[18]^. Biopsy analysis involves detecting epigenetic modifications, point mutations, translocations, amplifications, deletions, and assessing protein expression and phosphorylation [Figure 2]^[64]^. Functional assays using *in vitro* or *in vivo* models are employed to further validate the findings. Central to this approach is the detection of circRNAs, which act as stable, non-invasive biomarkers for tracking therapeutic resistance and guiding personalized cancer treatment decisions^[65]^. In NSCLC, the circRNA hsa_circ_0000190 and hsa_circ_0014235 were found to be upregulated in the plasma and sponge miRNAs, such as miR-142 and miR-124-3p, which promotes resistance to EGFR tyrosine kinase inhibitors^[65]^. In triple-negative breast cancer (TNBC), the circANKS1B found in serum contributed to the enhancement of EMT and paclitaxel resistance by targeting miR-148a-3p^[66]^. Likewise, circ_0005963 in plasma from colorectal cancer (CRC) enhances oxaliplatin resistance by governing glycolytic regulation through the GLUT1/miR-122 axis^[67]^. CircAKT3 is associated with gastric cancer targets and upregulates the autophagy pathway [PI3K/AKT/mechanistic target of rapamycin (mTOR)], helping promote cisplatin resistance^[68]^. In HCC, circMTO1 serves as a tumor suppressor but is downregulated in sorafenib resistance^[69]^. GBM-associated circRNAs, including circHIPK3 and circNT5E, have been detected in plasma and cerebrospinal fluid (CSF), with alterations in miRNA influencing TMZ resistance^[70]^. Similarly, circRNAs in ovarian (circCELSR1), pancreatic (circ-LDLRAD3), and bladder cancer (circRIP2) regulate pathways associated with chemotherapy resistance, including the EMT, PI3K/AKT, and transforming growth factor beta (TGF-β) signaling^[71]^. Overall, circRNAs from liquid biopsies are a novel source of non-invasive biomarkers for monitoring the development of drug resistance in cancer therapies in real time and offer the opportunity for strategic personalized therapeutic responses and interventions [Table 3]^[6]^. In the investigation of drug resistance, circRNAs have been implicated through several mechanisms. One of the primary roles of circRNAs is functioning as miRNA sponges - they sequester miRNAs that would otherwise inhibit oncogenic mRNA targets^[82]^. For example, circHIPK3 has been shown to sponge miR-124 to confer resistance to chemotherapeutic agents in colorectal and bladder cancers^[83]^. Likewise, in certain cancer subtypes, circRNA CDR1as can sequester miR-7, resulting in dysregulated EGFR expression and leading to resistance to targeted therapies in lung and breast cancers^[84]^. Beyond intravenously (IV) drug resistance mediated through the inhibition of miRNA activity, circRNAs can also affect protein targeting and transcription regulation, and some may even encode peptides related to multidrug resistance. These functions further support the role of circRNA as mediators in resistance pathways^[85]^. The relative ease of detecting cirRNAs through liquid biopsies - which provide a minimally invasive method to sample repeatedly and allow clinicians to track molecular signatures or emerging resistance over the course of treatment - offers a potential means of evaluating real-time treatment responses^[86]^. This is particularly important in advanced or later-stage cancers, where tumor heterogeneity and limited accessibility make repeated solid tissue biopsies impractical or impossible for traditional pathological diagnosis^[87]^. Current analyses of circulating circRNA focus on their expression patterns and their utility in inferring the internal molecular status and characteristics of the primary tumor. At the same time, these circulating circRNAs may serve as early indicators of emerging therapeutic resistance or treatment failure, enabling timely clinical intervention guided by rapid circRNA analysis^[88]^. Liquid biopsy markers offer non-invasive tools for monitoring drug resistance in cancer. ctDNA can identify genetic mutations and resistance-related alterations, but its low abundance in early-stage disease limits its sensitivity^[89]^. CTCs provide phenotypic and genotypic information, yet they are rare and technically challenging to isolate^[90]^. Exosomes, which carry proteins, DNA, and RNA, including circRNAs, offer both molecular stability and functional insights^[91]^. CircRNAs, due to their covalently closed structure, are highly stable and abundant in exosomes, making them promising biomarkers for real-time tracking of therapeutic resistance across various cancer types [Table 4]^[9]^.

**Table 3:** Components of liquid biopsy-based detection of circulating circRNAs for drug resistance monitoring in cancer

**S.N.**	**Cancer type**	**Liquid biomarker (circRNA)**	**Origin (body fluid)**	**Tendency in resistance**	**Target/pathway**	**Function/role**	**Ref.**
1	NSCLC	hsa_circ_0000190, hsa_circ_0014235	Plasma	Upregulated	miR-142, miR-124-3p	Promotes EGFR-TKI resistance by sponging miRNAs	[72]
2	TNBC	circ_0006528, circANKS1B	Serum	Upregulated	miR-148a-3p, miR-149-5p	Enhances EMT, metastasis and paclitaxel resistance	[73]
3	CRC	circ_0005963	Plasma	Upregulated	miR-122, GLUT1	Facilitates glycolysis and oxaliplatin resistance	[74]
4	Gastric cancer	circAKT3, circ_0000267	Plasma	Upregulated	PI3K/AKT/mTOR	Regulates cisplatin resistance via autophagy control	[75]
5	HCC	circ_0004913, circMTO1	Serum	Downregulated (in some)	miR-9, p21	Acts as tumor suppressor; associated with sorafenib resistance	[76]
6	Prostate cancer	circFOXO3	Plasma	Dysregulated	FOXO3 pathway	Modulates androgen deprivation therapy resistance	[77]
7	Pancreatic cancer	circ-LDLRAD3	Plasma	Upregulated	miR-137	Promotes gemcitabine resistance via EMT regulation	[78]
8	GBM	circHIPK3, circNT5E	CSF/plasma	Upregulated	miR-124, miR-422a	Enhances proliferation and TMZ resistance	[79]
9	Ovarian cancer	circCELSR1	Plasma	Upregulated	miR-1252, FOXR2	Contributes to cisplatin resistance via PI3K/AKT activation	[80]
10	Bladder cancer	circRIP2	Urine/plasma	Upregulated	miR-1305, TGF-β2	Facilitates EMT and chemoresistance	[81]

circRNAs: Circular RNAs; NSCLC: non-small cell lung cancer; EGFR-TKI: epidermal growth factor receptor-tyrosine kinase inhibitor; miRNAs: microRNAs; TNBC: triple-negative breast cancer; EMT: epithelial-mesenchymal transition; CRC: colorectal cancer; PI3K: phosphoinositide 3-kinase; AKT: protein kinase B; mTOR: mechanistic target of rapamycin; HCC: hepatocellular carcinoma; FOXO3: forkhead box O3; GBM: glioblastoma; CSF: cerebrospinal fluid; TMZ: temozolomide; FOXR2: forkhead box R2; TGF-β2: transforming growth factor beta 2.

**Table 4:** Comparison of liquid biopsy markers for drug resistance surveillance in cancer

**S.N.**	**Marker type**	**Biological source**	**Detection of circRNAs**	**Utility in drug resistance monitoring**	**Advantages**	**Limitations**	**Ref.**
1	ctDNA	Plasma/serum	Not applicable (DNA only)	Detects resistance mutations (e.g., EGFR, KRAS)	Non-invasive; mutation-specific tracking; quick turnaround	Does not reflect non-coding RNA regulation; low abundance in early-stage disease	[92]
2	CTCs	Whole blood	Possible (but rarely used for circRNA)	Phenotypic and genotypic profiling of resistance-related pathways	Can be cultured; provides both RNA and protein information	Low frequency; difficult to isolate and purify; not ideal for circRNA analysis	[86]
3	Exosomes	Plasma, serum, saliva, urine, CSF	Highly enriched for circRNAs	Reflects tumor-secreted resistance-related molecules and intercellular signals	Protect circRNAs from degradation; tumor-specific; suitable for dynamic tracking	Isolation and characterization methods not yet standardized; requires optimization	[91]
4	cfRNA	Plasma/serum	Includes circRNAs (after enrichment)	Tracks therapy-related changes in gene expression and resistance regulation	Minimally invasive; suitable for repeated monitoring	CircRNAs are a minor fraction of total cfRNA; requires enrichment and sensitive detection	[93]
5	Exosomal circRNAs	Exosome fraction from plasma/serum	Direct and stable source of circRNAs	Dynamic monitoring of resistance; potential predictive biomarker	High specificity and stability; resistant to RNases	Requires high-quality isolation; functional validation needed	[94]

circRNAs: Circular RNAs; ctDNA: circulating tumor DNA; EGFR: epidermal growth factor receptor; KRAS: kirsten rat sarcoma viral oncogene homolog; CTCs: circulating tumor cells; CSF: cerebrospinal fluid; cfRNA: cell-free RNA.

## TOOLS AND TECHNIQUES FOR MONITORING LIQUID BIOPSY OF CIRCRNAS IN CANCER THERAPY RESISTANCE

Monitoring of circulating circRNAs by liquid biopsy utilizes various RNA detection methods, including qRT-PCR, ddPCR, and RNA-seq^[94,95]^. qRT-PCR is a sensitive, rapid technique, ddPCR enables absolute quantification, and RNA-seq provides comprehensive circRNA profiling. These methods differ in cost, throughput, and suitability for clinical application^[96]^. Non-invasive monitoring of circRNAs in circulation to track mechanisms of therapeutic resistance requires a multifaceted approach combining molecular techniques with bioinformatics methods to provide real-time insight into the biology of tumors^[97,98]^. Blood should be collected using RNase-free ethylenediaminetetraacetic acid (EDTA) tubes and processed quickly by centrifugation to provide plasma. Exosome preparations, whether obtained by ultracentrifugation or polystyrene-based commercial systems, can provide tumor-specific circRNAs, further increasing their utility for monitoring tumor evolution in the context of therapy^[99,100]^. High-sensitivity kits provide total RNA extraction that can be treated with RNase R to enrich circRNAs by removing linear RNA^[101]^. Finally, divergent primers can be developed for the qRT-PCR method to detect known circRNAs that can be isolated with ddPCR^[102]^. High-sensitivity droplet dPCR provides sensitive absolute quantitation of circRNAs as a complementary method for potentially longitudinal monitoring and/or resistance contributions^[103]^.

**Figure 3:**
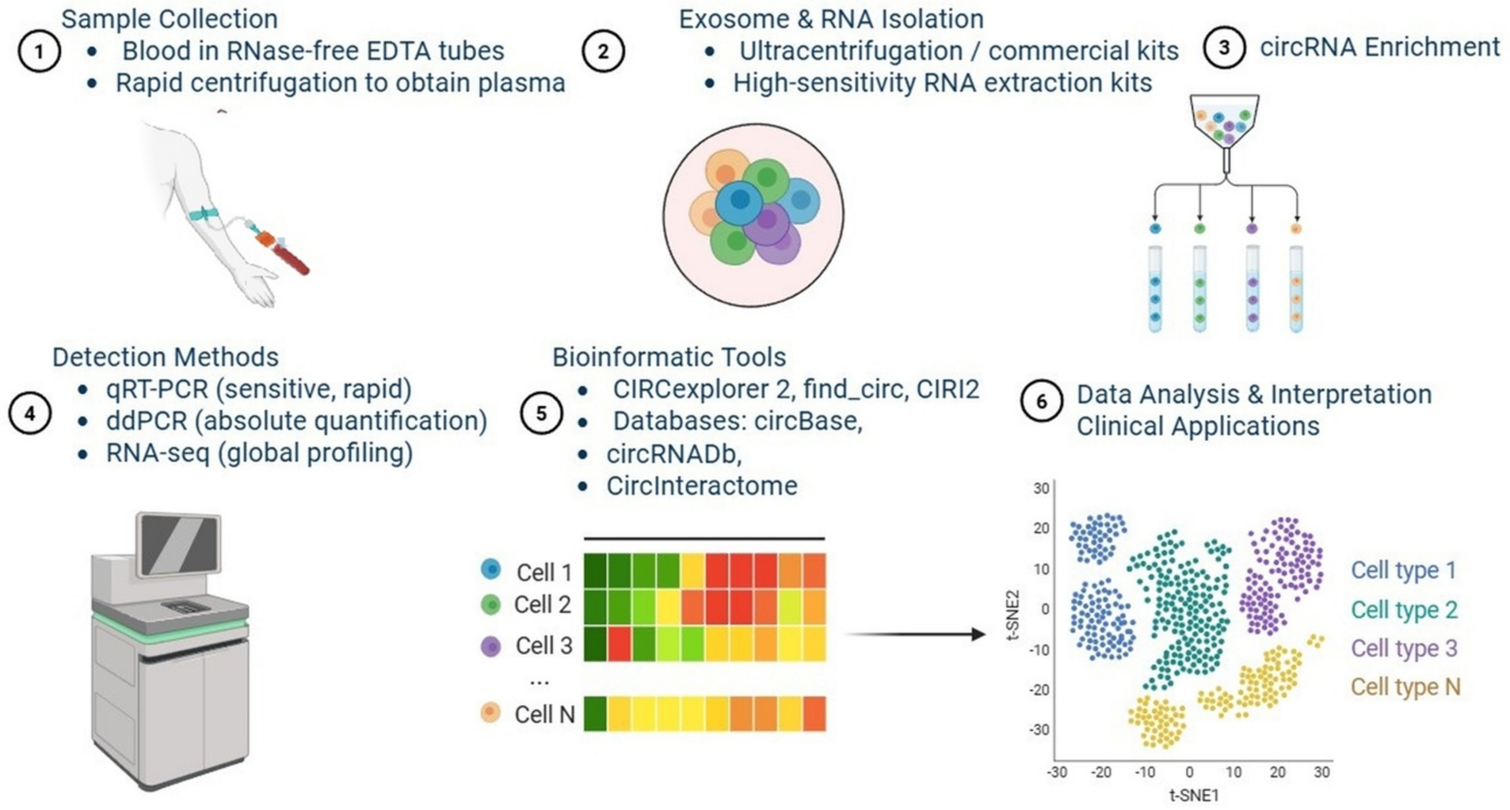
Schematic representation of the experimental and analytical pipeline for circRNA detection and characterization. (1) Sample collection: blood is collected in RNase-free EDTA tubes, followed by rapid centrifugation to obtain plasma; (2) Exosome and RNA isolation: performed using ultracentrifugation or commercial high-sensitivity RNA extraction kits; (3) CircRNA enrichment: strategies to enrich circRNAs for downstream analyses; (4) Detection methods: include qRT-PCR (sensitive, rapid), ddPCR (absolute quantification), and RNA-seq (global profiling); (5) Bioinformatic tools: circRNA analysis pipelines such as CIRCexplorer2, find_circ, and CIRI2, along with databases such as circBase, circRNADb, and CircInteractome; (6) Data analysis and interpretation: application of circRNA data in clinical translation and biomarker development [Created in BioRender. Singh DD (2025)]. circRNA: Circular RNA; EDTA: ethylenediaminetetraacetic acid; qRT-PCR: quantitative reverse transcription polymerase chain reaction; ddPCR: droplet digital polymerase chain reaction; RNA-seq: RNA sequencing.

The combination of circRNA-specific back-splice junction detection with deconvolution-based methods improves specificity, which would enable the identification of cancer type- or drug resistance-associated circRNAs at low abundance. Deconvoluted sequencing in combination with longitudinal RNA-seq also facilitates early detection of therapeutic resistance^[101]^. Future integration of deconvoluted sequencing with artificial intelligence (AI) and single-cell RNA-seq libraries is expected to further improve sensitivity and predictive power, and to support the use of bulk RNA-seq as a non-invasive monitoring tool^[101-104]^. As RNA-seq becomes more affordable, global profiling of circRNAs may be achieved through the use of rRNA-depleted or RNase R-treated libraries and bioinformatics tools such as CIRCexplorer2, find_circ and CIRI2^[105]^. Exosome-derived circRNAs also display tumor specificity, as drug-resistant tumor cells tend to preferentially package them, which could provide insight into the persistence and localization of drug resistance during therapy^[106]^. The integration of bioinformatics platforms and databases (circBase, circRNADb, and CircInteractome) will facilitate circRNA annotation, differential expression analysis, and functional characterization, while incorporating pathway and network analysis platforms will help elucidate the mechanisms by which circRNAs contribute to cancer drug resistance [Figure 3]^[107]^.

The integration of these tools enables the development of dynamic circRNA-based biomarker panels that can provide real-time guidance for therapeutic decisions^[95]^. Although these approaches are still evolving, challenges remain in standardizing sample handling, accounting for pre-analytical variability, and differentiating tumor-derived circRNAs from those originating from non-malignant sources^[16]^. Nevertheless, the convergence of effective detection methods, sophisticated analytical methods, and key clinical investigations is positioning circRNA profiling as a key part of future precision oncology^[96]^. Looking ahead, liquid biopsies leveraging circRNAs to monitor therapeutic resistance are expected to increasingly support personalized, adaptive cancer treatment plans^[2]^. A comparison of RNA detection methods is presented in Table 5.

**Table 5:** Comparison of RNA detection methods

**S.N.**	**RNA detection method**	**Technology/platform**	**Advantages**	**Disadvantages**	**Ref.**
1	qRT-PCR	Fluorescence-based amplification (SYBR/Probe)	High sensitivity and specificity; cost-effective; fast turnaround; widely used for known targets	Requires prior knowledge of RNA; limited multiplexing; not ideal for novel discovery	[97]
2	ddPCR	Droplet partitioning + fluorescence detection	Absolute quantification; extremely sensitive; useful for low-abundance RNAs; high reproducibility	Higher cost; requires specialized equipment; limited throughput	[98]
3	Conventional RT-PCR	End-point PCR + gel electrophoresis	Simple and inexpensive; good for qualitative confirmation	Low sensitivity; not quantitative; risk of contamination	[99]
4	RNA-seq	NGS	Unbiased transcriptome-wide profiling; can detect novel RNAs including circRNAs; enables differential expression analysis	High cost; requires advanced bioinformatics; longer turnaround	[100]
5	Microarrays	Hybridization-based array platforms	Enable profiling of many RNAs at once; established technology	Limited to known sequences; lower dynamic range and sensitivity than RNA-seq	[101]
6	Northern blotting	RNA separation by gel + probe hybridization	Provides RNA size and abundance information; visual confirmation	Low sensitivity; labor-intensive; requires large RNA input	[102]
7	ISH	Tissue-based hybridization with labeled probes	Detects spatial localization of RNA in tissue or cells	Low throughput; semi-quantitative; needs tissue sections	[103]
7	NanoString nCounter	Barcoded probe-based digital counting	No need for amplification; highly multiplexed; works with degraded samples	Expensive; limited to pre-designed probes; not suitable for discovery	[104]

qRT-PCR: Quantitative reverse transcription polymerase chain reaction; SYBR: synergy of selective binding reagents; ddPCR: droplet digital polymerase chain reaction; RNA-seq: RNA sequencing; NGS: next-generation sequencing; ISH: *in situ* hybridization.

## CLINICAL TRIALS ON LIQUID BIOPSY-BASED DETECTION OF CIRCULATING CIRCRNAS FOR TRACKING DRUG RESISTANCE IN CANCER

Various preclinical and translational studies have demonstrated that circRNAs such as circFOXO3, circHIPK3, and F-circEA are involved in drug resistance pathways, including the evasion of apoptosis, regulation of autophagy, and drug efflux [Table 6]^[105]^. Moreover, trials involving liquid biopsies have concentrated on ctDNA, CTCs, or miRNAs to monitor treatment response or early resistance in cancers such as breast, lung, and CRC^[106]^. For example, ctDNA-based clinical trials, including SERENA-6 by AstraZeneca and the NHS London pilot, have demonstrated the utility of early mutation detection in guiding therapy switches^[107]^. In contrast, circRNAs have not yet been integrated into routine clinical workflows^[52]^. Efforts have been made in observational studies and small cohort studies to study circRNAs in biofluids (especially in exosomes) as indicators of drug resistance in breast, prostate, and NSCLCs^[111]^. Importantly, these earliest studies provide proof-of-concept evidence that circRNA profiles can capture the dynamic evolution of a tumor’s adaptation to therapy^[24]^. Given the advances in sequencing and computational tools available for back-splice junction recognition, detecting circRNAs in clinical samples is technically feasible^[112]^. To translate these findings into clinical practice, dedicated trials are needed to evaluate the sensitivity, specificity, and predictive value of circRNA panels in monitoring drug resistance^[113]^. Future clinical investigations should focus on validating circRNA signatures in longitudinal patient samples, combining ctDNA and circRNA assays, and gradually integrating circRNAs into companion diagnostic workflows. Conducting such trials would represent an important milestone in personalized oncology, enabling real-time therapy adjustments based on evolving resistance patterns identified non-invasively^[6]^. Achieving this will require multi-center collaboration, standardized protocols for circRNA extraction and quantification, and regulatory support for biomarker-based decision making. As precision oncology advances, circRNA liquid biopsies represent a promising tool for early resistance detection and optimized therapy across multiple cancer types.

**Table 6:** Summary of clinical and translational insights related to circRNA-based liquid biopsy for drug resistance monitoring

**S.N.**	**Trial/study name**	**Status**	**Target cancer type**	**Liquid biopsy marker**	**CircRNA focus**	**Detection method**	**Purpose/outcome**	**Ref.**
1	F-circEA study	Completed (translational)	NSCLC	Plasma cfRNA/exosomes	F-circEA (from EML4-ALK)	qRT-PCR	Proof-of-concept: F-circEA detected in the plasma of ALK-positive patients; shows biomarker potential	[108]
2	CircRNA microarray study	Translational	Ovarian cancer	Plasma circRNAs	Multiple novel circRNAs	CircRNA microarray, RT-PCR	Identified differentially expressed circRNAs related to drug sensitivity	[109]
3	Pancreatic cancer circRNA panel study	Translational	PDAC	Plasma	5-CircRNA diagnostic panel	RNA-seq + qRT-PCR	Proposed a diagnostic + drug response circRNA signature; preclinical stage	[95]
4	ctDNA-Guided SERENA-6 trial (AstraZeneca)	Phase III	Breast cancer	ctDNA	Not circRNA-specific	ddPCR/NGS	Demonstrated ctDNA-guided treatment switching improved PFS; circRNA potential underexplored	[107]
4	NHS London liquid biopsy pilot	Pilot program	Breast cancer	ctDNA	Not circRNA-specific	ddPCR	Applied ctDNA for ESR1 mutation detection; supports framework for circRNA integration	[110]

circRNA: Circular RNA; NSCLC: non-small cell lung cancer; cfRNA: cell-free RNA; EML4-ALK: echinoderm microtubule-associated protein-Like 4 – anaplastic lymphoma kinase; qRT-PCR: quantitative reverse transcription polymerase chain reaction; PDAC: pancreatic ductal adenocarcinoma; RNA-seq: RNA sequencing; ctDNA: circulating tumor DNA; ddPCR: droplet digital polymerase chain reaction; NGS: next-generation sequencing; PFS: progression-free survival; ESR1: estrogen receptor 1.

## LIMITATIONS OF THE STUDY ON CLINICAL TRIALS ON LIQUID BIOPSY-BASED DETECTION OF CIRCULATING CIRCRNAS FOR TRACKING DRUG RESISTANCE IN CANCER

Despite the growing interest in utilizing liquid biopsy for cancer management, clinical trials specifically focused on the detection of circulating circRNAs for monitoring drug resistance remain limited and face several important challenges^[114]^. One of the foremost limitations is the lack of ongoing or completed large-scale clinical trials that investigate the diagnostic or prognostic utility of circRNAs in a real-world therapeutic context^[115]^. Most available studies are preclinical or translational, primarily using *in vitro* cell lines or small patient cohorts, which limits their generalizability and clinical applicability^[116]^. The lack of robust clinical validation and longitudinal patient data makes it difficult to establish circRNAs as reliable biomarkers for tracking drug resistance. Additionally, the standardization of circRNA detection protocols presents a major barrier^[117]^. There is currently no universally accepted method for the enrichment, isolation, and quantification of circRNAs in clinical samples^[118]^. Techniques such as RNase R treatment, divergent primer design, and RNA seq are highly sensitive to experimental conditions, leading to variability in results across laboratories and studies^[119]^. Furthermore, the low abundance and tissue-specific expression of circRNAs often require advanced technologies such as ddPCR or deep RNA seq, which may not be available in routine diagnostic settings. Another major limitation lies in the functional characterization of identified circRNAs^[52]^. While many studies report associations between specific circRNAs and drug resistance phenotypes, few have established causal mechanisms or validated these findings *in vivo*^[113]^. This gap undermines the translational potential of circRNA signatures and limits their incorporation into clinical trial designs^[120]^. Moreover, circRNAs often function as competing endogenous RNAs or miRNA sponges, and their network-level interactions with other non-coding RNAs or mRNAs are complex and still not fully understood^[84]^. From a regulatory and ethical standpoint, the introduction of novel biomarkers into clinical trials necessitates rigorous validation under Good Clinical Practice and compliance with data safety and ethical standards, which adds time and logistical complexity to trial initiation and conduct^[121]^. Additionally, the cost and data burden of high-throughput circRNA profiling, especially when integrated with other omics data, may limit its widespread adoption, particularly in low-resource clinical settings^[122]^.

Another issue is the lack of clear clinical utility or guidelines on how circRNA-based resistance detection would influence therapeutic decisions^[123]^. Unlike ctDNA, which can reveal actionable mutations leading to drug switching [e.g., estrogen receptor 1 (ESR1) mutations in breast cancer], circRNAs currently lack such validated clinical pathways^[124]^. Their integration into decision-making algorithms and treatment protocols remains theoretical at this stage. Lastly, patient heterogeneity in terms of tumor type, treatment history, and genetic background adds another layer of complexity, making it difficult to define universal circRNA panels for resistance tracking^[125]^. In conclusion, while the scientific foundation for using circulating circRNAs as non-invasive biomarkers for drug resistance is strong, the absence of clinical trial validation, methodological standardization, and real-time clinical utility assessment significantly limits current implementation. Addressing these limitations through collaborative, multi-center clinical trials and robust bioinformatics pipelines will be crucial for advancing the role of circRNAs in precision oncology.

## FUTURE PROSPECTIVE

The significant potential of circRNAs in liquid biopsy for monitoring cancer drug resistance necessitates extensive future research, both experimental and clinical^[44]^. To begin, large-scale multi-center clinical trials should be launched to evaluate the clinical utility of circRNAs as predictive biomarkers of therapeutic resistance in multiple cancer types^[112]^. These trials should assess circRNA expression levels in solid tumors, hematological malignancies, and pediatric hematological malignancies using a longitudinal model^[126]^. The expression profiles will be correlated with resistance, progression-free survival (PFS), and therapeutic response to treatment modifications^[120]^. To ensure consistency and reproducibility, standardized pre-analytical and analytical methods are essential^[44]^. This includes establishing global standards for sample collection, circRNA extraction, RNase R-based enrichment, and detection using highly sensitive techniques (e.g., qRT-PCR, ddPCR, or RNA seq)^[52]^. Long-term data on circRNA expression will further enhance the clinical translatability of circRNAs as predictive biomarkers of therapeutic resistance, with far-reaching implications in cancer management.

The development of affordable, point-of-care diagnostic platforms for detecting tumor-specific circRNAs in body fluids could enable real-time monitoring of resistance while facilitating personalized treatment planning^[127]^. Future work should also prioritize functional characterization of resistance-associated circRNAs, including their molecular roles and interactions with miRNAs, mRNAs, and signaling pathways^[85]^. Such efforts would establish the biomarker potential of circRNAs and support the feasibility of therapeutically targeting oncogenic circRNAs. Combining circRNA profiles with other omics data (e.g., proteomics, genomics, metabolomics), using AI and machine learning approaches, may further enhance predictive accuracy and facilitate the development of comprehensive resistance signatures^[128]^. Another important step will be advancing circRNAs as companion diagnostics to guide precision-targeted therapies or immunotherapies^[129]^. Close collaboration with regulatory authorities will also be critical to define the biomarker qualification pathway for circRNAs while ensuring compliance with clinical and ethical standards^[130]^. In summary, advancing the field of circRNAs in liquid biopsy requires a multidisciplinary strategy that integrates molecular biology, bioinformatics, clinical oncology, and regulatory science. With sustained research investment and a strong translational focus, circRNAs hold the promise to transform non-invasive cancer monitoring and the precision management of therapeutic resistance.

## CONCLUSION

Combining liquid biopsy with circRNA research is a revolutionary way to monitor drug resistance in cancer. circRNAs have a covalently closed-loop structure, making them highly stable in circulation and ideal candidates for non-invasive biomarkers in real-time disease monitoring. Advances in molecular testing such as qRT-PCR, ddPCR, and RNA seq have provided adequate sensitivity and specificity to detect circRNA from plasma, serum, and exosomes. In this way, circRNA can be linked to specific resistance mechanisms that include the regulation of apoptosis, autophagy, epithelial–mesenchymal transition, and drug efflux. By assessing therapeutic responses, circRNA-based liquid biopsy can capture molecular changes, enabling real-time interpretation of these dynamic events. Beyond this analytical purpose, liquid biopsy with circRNAs can guide treatment decisions, enable early detection of resistance, and improve personalized cancer care. Further standardization and clinical validation of such approaches are needed; nonetheless, current evidence supports the potential of circulating circRNAs as future clinical tools in precision oncology.

## DECLARATIONS

### Copyright

© The Author(s) 2025.

## References

[1] Wang X, Zhang H, Chen X (2019). Drug resistance and combating drug resistance in cancer. Cancer Drug Resist.

[2] Ma L, Guo H, Zhao Y (2024). Liquid biopsy in cancer current: status, challenges and future prospects. Signal Transduct Target Ther.

[3] Huang L, Rong Y, Tang X, Yi K, Wu J, Wang F (2021). Circular RNAs are promising biomarkers in liquid biopsy for the diagnosis of non-small cell lung cancer. Front Mol Biosci.

[4] Batool SM, Yekula A, Khanna P (2023). The liquid biopsy consortium: challenges and opportunities for early cancer detection and monitoring. Cell Rep Med.

[5] Wen G, Zhou T, Gu W (2021). The potential of using blood circular RNA as liquid biopsy biomarker for human diseases. Protein Cell.

[6] Zhang Y, Wang Y, Su X, Wang P, Lin W (2021). The value of circulating circular RNA in cancer diagnosis, monitoring, prognosis, and guiding treatment. Front Oncol.

[7] Wang S, Zhang K, Tan S (2021). Circular RNAs in body fluids as cancer biomarkers: the new frontier of liquid biopsies. Mol Cancer.

[8] Babin L, Andraos E, Fuchs S, Pyronnet S, Brunet E, Meggetto F (2021). From circRNAs to fusion circRNAs in hematological malignancies. JCI Insight.

[9] Hua J, Wang Z, Cheng X, Dai J, Zhao P (2025). Circular RNAs modulate cancer drug resistance: advances and challenges. Cancer Drug Resist.

[10] Wu X, Shi M, Lian Y, Zhang H (2023). Exosomal circRNAs as promising liquid biopsy biomarkers for glioma. Front Immunol.

[11] Garlapati P, Ling J, Chiao PJ, Fu J (2021). Circular RNAs regulate cancer-related signaling pathways and serve as potential diagnostic biomarkers for human cancers. Cancer Cell Int.

[12] Choi SS, Kim SE, Oh SY, Ahn YH (2022). Clinical implications of circulating circular RNAs in lung cancer. Biomedicines.

[13] Li F, Yang Q, He AT, Yang BB (2021). Circular RNAs in cancer: limitations in functional studies and diagnostic potential. Semin Cancer Biol.

[14] Ghani MU, Du L, Moqbel AQ (2025). Exosomal ncRNAs in liquid biopsy: a new paradigm for early cancer diagnosis and monitoring. Front Oncol.

[15] Liu XY, Zhang Q, Guo J (2021). The role of circular RNAs in the drug resistance of cancers. Front Oncol.

[16] Kundu I, Varshney S, Karnati S, Naidu S (2024). The multifaceted roles of circular RNAs in cancer hallmarks: from mechanisms to clinical implications. Mol Ther Nucleic Acids.

[17] Zhang Q, Wang W, Zhou Q (2020). Roles of circRNAs in the tumour microenvironment. Mol Cancer.

[18] Liu W, Niu J, Huo Y (2025). Role of circular RNAs in cancer therapy resistance. Mol Cancer.

[19] Rochow H, Franz A, Jung M (2020). Instability of circular RNAs in clinical tissue samples impairs their reliable expression analysis using RT-qPCR: from the myth of their advantage as biomarkers to reality. Theranostics.

[20] Radanova M, Mihaylova G, Tasinov O (2021). New circulating circular RNAs with diagnostic and prognostic potential in advanced colorectal cancer. Int J Mol Sci.

[21] Bersani F, Picca F, Morena D (2023). Exploring circular MET RNA as a potential biomarker in tumors exhibiting high MET activity. J Exp Clin Cancer Res.

[22] Roy S, Kanda M, Nomura S (2022). Diagnostic efficacy of circular RNAs as noninvasive, liquid biopsy biomarkers for early detection of gastric cancer. Mol Cancer.

[23] Lu S, Liang Y, Li L (2023). Inferring circRNA-drug sensitivity associations via dual hierarchical attention networks and multiple kernel fusion. BMC Genomics.

[24] Pisignano G, Michael DC, Visal TH, Pirlog R, Ladomery M, Calin GA (2023). Going circular: history, present, and future of circRNAs in cancer. Oncogene.

[25] Wilusz JE (2017). Circular RNAs: unexpected outputs of many protein-coding genes. RNA Biol.

[26] Liu X, Zhang Y, Zhou S, Dain L, Mei L, Zhu G (2022). Circular RNA: an emerging frontier in RNA therapeutic targets, RNA therapeutics, and mRNA vaccines. J Control Release.

[27] Nielsen AF, Bindereif A, Bozzoni I (2022). Best practice standards for circular RNA research. Nat Methods.

[28] Li Q, Wang Y, Wu S (2019). CircACC1 regulates assembly and activation of AMPK complex under metabolic stress. Cell Metab.

[29] Hussen BM, Abdullah SR, Jaafar RM (2025). Circular RNAs as key regulators in cancer hallmarks: new progress and therapeutic opportunities. Crit Rev Oncol Hematol.

[30] Yang Q, Li F, He AT, Yang BB (2021). Circular RNAs: expression, localization, and therapeutic potentials. Mol Ther.

[31] Zhou M, Xiao MS, Li Z, Huang C (2021). New progresses of circular RNA biology: from nuclear export to degradation. RNA Biol.

[32] Hashemi M, Khosroshahi EM, Daneii P (2025). Emerging roles of CircRNA-miRNA networks in cancer development and therapeutic response. Noncoding RNA Res.

[33] Wei Z, Shi Y, Xue C (2022). Understanding the dual roles of circHIPK3 in tumorigenesis and tumor progression. J Cancer.

[34] Singh DD, Yadav DK, Shin D (2025). Non-coding RNAs in cancer therapy-induced cardiotoxicity: unlocking precision biomarkers for early detection. Cell Signal.

[35] Singh DD, Kim Y, Choi SA, Han I, Yadav DK (2023). Clinical significance of microRNAs, long non-coding RNAs, and circRNAs in cardiovascular diseases. Cells.

[36] Liu YY, Zhang LY, Du WZ (2019). Circular RNA circ-PVT1 contributes to paclitaxel resistance of gastric cancer cells through the regulation of ZEB1 expression by sponging miR-124-3p. Biosci Rep.

[37] Xue C, Li G, Lu J, Li L (2021). Crosstalk between circRNAs and the PI3K/AKT signaling pathway in cancer progression. Signal Transduct Target Ther.

[38] Zhao M, Lin M, Zhang Z, Ye L (2025). Research progress of circular RNA FOXO3 in diseases (review). Glob Med Genet.

[39] Shao Y, Song Y, Xu S, Li S, Zhou H (2020). Expression profile of circular RNAs in oral squamous cell carcinoma. Front Oncol.

[40] Yan T, Tian X, Liu F (2022). The emerging role of circular RNAs in drug resistance of non-small cell lung cancer. Front Oncol.

[41] Chen J, Yang J, Fei X, Wang X, Wang K (2021). CircRNA ciRS-7: a novel oncogene in multiple cancers. Int J Biol Sci.

[42] Han D, Li J, Wang H (2017). Circular RNA circMTO1 acts as the sponge of microRNA-9 to suppress hepatocellular carcinoma progression. Hepatology.

[43] Xia X, Li X, Li F (2019). A novel tumor suppressor protein encoded by circular AKT3 RNA inhibits glioblastoma tumorigenicity by competing with active phosphoinositide-dependent Kinase-1. Mol Cancer.

[44] Xu T, Wang M, Jiang L (2020). CircRNAs in anticancer drug resistance: recent advances and future potential. Mol Cancer.

[45] Zhang J, Luo Z, Zheng Y, Duan M, Qiu Z, Huang C (2024). CircRNA as an Achilles heel of cancer: characterization, biomarker and therapeutic modalities. J Transl Med.

[46] Adhit KK, Wanjari A, Menon S, Siddhaarth K (2023). Liquid biopsy: an evolving paradigm for non-invasive disease diagnosis and monitoring in medicine. Cureus.

[47] Wang M, Yu F, Li P, Wang K (2020). Emerging function and clinical significance of exosomal circRNAs in cancer. Mol Ther Nucleic Acids.

[48] Martinez-Dominguez MV, Zottel A, Šamec N (2021). Current technologies for RNA-directed liquid diagnostics. Cancers.

[49] Wang K, Bai X, Xue Y (2023). Absolute quantification of circRNA using digital reverse transcription-hyperbranched rolling circle amplification. Sens Actuators B Chem.

[50] Masante L, Susin G, Baudet M

[51] Bauer-Negrini G, Cordenonsi da Fonseca G, Gottfried C, Herbert J (2022). Usability evaluation of circRNA identification tools: development of a heuristic-based framework and analysis. Comput Biol Med.

[52] Gaffo E, Buratin A, Dal Molin A, Bortoluzzi S (2022). Sensitive, reliable and robust circRNA detection from RNA-seq with CirComPara2. Brief Bioinform.

[53] Chen L, Wang C, Sun H (2021). The bioinformatics toolbox for circRNA discovery and analysis. Brief Bioinform.

[54] Zhang N, Wang X, Li Y (2025). Mechanisms and therapeutic implications of gene expression regulation by circRNA-protein interactions in cancer. Commun Biol.

[55] Velpula T, Buddolla V (2025). Enhancing detection and monitoring of circulating tumor cells: integrative approaches in liquid biopsy advances. J Liq Biopsy.

[56] Ren F, Fei Q, Qiu K, Zhang Y, Zhang H, Sun L (2024). Liquid biopsy techniques and lung cancer: diagnosis, monitoring and evaluation. J Exp Clin Cancer Res.

[57] Gao Y, Li C, Ji T, Yu K, Gao X (2025). The biological function and mechanism of action of circRNA as a potential target in colorectal cancer. Crit Rev Oncol Hematol.

[58] Zhang Z, Yang T, Xiao J (2018). Circular RNAs: promising biomarkers for human diseases. EBioMedicine.

[59] Connal S, Cameron JM, Sala A (2023). Liquid biopsies: the future of cancer early detection. J Transl Med.

[60] Feng XY, Zhu SX, Pu KJ, Huang HJ, Chen YQ, Wang WT (2023). New insight into circRNAs: characterization, strategies, and biomedical applications. Exp Hematol Oncol.

[61] Alimohammadi M, Kahkesh S, Khoshnazar SM (2025). Circular RNAs and doxorubicin resistance in cancer: molecular mechanisms and potential treatment targets. Gene.

[62] Siavashy S, Soltani M, Rahimi S, Hosseinali M, Guilandokht Z, Raahemifar K (2024). Recent advancements in microfluidic-based biosensors for detection of genes and proteins: applications and techniques. Biosens Bioelectron X.

[63] Latifi-Pakdehi T, Khezrian A, Doosti-Irani A, Afshar S, Mahdavinezhad A (2024). Investigating the biomarker value of circRNAs in the diagnosis of colorectal cancer: a systematic review. Discov Oncol.

[64] Rashid S, Sun Y, Ali Khan Saddozai U (2024). Circulating tumor DNA and its role in detection, prognosis and therapeutics of hepatocellular carcinoma. Chin J Cancer Res.

[65] Luo YH, Yang YP, Chien CS (2021). Circular RNA hsa_circ_0000190 facilitates the tumorigenesis and immune evasion by upregulating the expression of soluble PD-L1 in non-small-cell lung cancer. Int J Mol Sci.

[66] Zeng K, He B, Yang BB (2018). The pro-metastasis effect of circANKS1B in breast cancer. Mol Cancer.

[67] Wang X, Zhang H, Yang H (2020). Exosome-delivered circRNA promotes glycolysis to induce chemoresistance through the miR-122-PKM2 axis in colorectal cancer. Mol Oncol.

[68] Huang X, Li Z, Zhang Q (2019). Circular RNA AKT3 upregulates PIK3R1 to enhance cisplatin resistance in gastric cancer via miR-198 suppression. Mol Cancer.

[69] Dong ZR, Ke AW, Li T (2021). CircMEMO1 modulates the promoter methylation and expression of TCF21 to regulate hepatocellular carcinoma progression and sorafenib treatment sensitivity. Mol Cancer.

[70] Salami R, Salami M, Mafi A, Vakili O, Asemi Z (2022). Circular RNAs and glioblastoma multiforme: focus on molecular mechanisms. Cell Commun Signal.

[71] Weidle UH, Birzele F (2024). Deregulated circRNAs in epithelial ovarian cancer with activity in preclinical *in vivo* models: identification of targets and new modalities for therapeutic intervention. Cancer Genomics Proteomics.

[72] Zhu Q, Zhang Y, Li M (2023). MiR-124-3p impedes the metastasis of non-small cell lung cancer via extracellular exosome transport and intracellular PI3K/AKT signaling. Biomark Res.

[73] Xu A, Zhu L, Yao C, Zhou W, Guan Z (2024). The therapeutic potential of circular RNA in triple-negative breast cancer. Cancer Drug Resist.

[74] Li T, Wang WC, McAlister V, Zhou Q, Zheng X (2021). Circular RNA in colorectal cancer. J Cell Mol Med.

[75] Cai X, Nie J, Chen L, Yu F (2020). Circ_0000267 promotes gastric cancer progression via sponging MiR-503-5p and regulating HMGA2 expression. Mol Genet Genomic Med.

[76] Han D, Li J, Wang H (2017). Circular RNA circMTO1 acts as the sponge of microRNA-9 to suppress hepatocellular carcinoma progression. Hepatology.

[77] Kong Z, Wan X, Lu Y (2020). Circular RNA circFOXO3 promotes prostate cancer progression through sponging miR-29a-3p. J Cell Mol Med.

[78] Yang F, Liu DY, Guo JT (2017). Circular RNA circ-LDLRAD3 as a biomarker in diagnosis of pancreatic cancer. World J Gastroenterol.

[79] Morena D, Picca F, Taulli R (2019). CircNT5E/miR-422a: a new circRNA-based ceRNA network in glioblastoma. Transl Cancer Res.

[80] Zeng XY, Yuan J, Wang C (2020). circCELSR1 facilitates ovarian cancer proliferation and metastasis by sponging miR-598 to activate BRD4 signals. Mol Med.

[81] Su Y, Feng W, Shi J, Chen L, Huang J, Lin T (2020). circRIP2 accelerates bladder cancer progression via miR-1305/Tgf-β2/smad3 pathway. Mol Cancer.

[82] Zhu J, Li Q, Wu Z, Xu W, Jiang R (2024). Circular RNA-mediated miRNA sponge & RNA binding protein in biological modulation of breast cancer. Noncoding RNA Res.

[83] Kai D, Yannian L, Yitian C, Dinghao G, Xin Z, Wu J (2018). Circular RNA HIPK3 promotes gallbladder cancer cell growth by sponging microRNA-124. Biochem Biophys Res Commun.

[84] Huang Y, Zhang C, Xiong J, Ren H (2021). Emerging important roles of circRNAs in human cancer and other diseases. Genes Dis.

[85] He AT, Liu J, Li F, Yang BB (2021). Targeting circular RNAs as a therapeutic approach: current strategies and challenges. Signal Transduct Target Ther.

[86] Lone SN, Nisar S, Masoodi T (2022). Liquid biopsy: a step closer to transform diagnosis, prognosis and future of cancer treatments. Mol Cancer.

[87] Hirahata T, Ul Quraish R, Quraish AU, Ul Quraish S, Naz M, Razzaq MA (2022). Liquid biopsy: a distinctive approach to the diagnosis and prognosis of cancer. Cancer Inform.

[88] Verduci L, Strano S, Yarden Y, Blandino G (2019). The circRNA-microRNA code: emerging implications for cancer diagnosis and treatment. Mol Oncol.

[89] Wang H, Zhang Y, Zhang H (2024). Liquid biopsy for human cancer: cancer screening, monitoring, and treatment. MedComm.

[90] Lin D, Shen L, Luo M (2021). Circulating tumor cells: biology and clinical significance. Signal Transduct Target Ther.

[91] Shi X, Wang B, Feng X, Xu Y, Lu K, Sun M (2020). circRNAs and exosomes: a mysterious frontier for human cancer. Mol Ther Nucleic Acids.

[92] Ge Q, Zhang ZY, Li SN, Ma JQ, Zhao Z (2024). Liquid biopsy: comprehensive overview of circulating tumor DNA (Review). Oncol Lett.

[93] Zhong P, Bai L, Hong M (2024). A comprehensive review on circulating cfRNA in plasma: implications for disease diagnosis and beyond. Diagnostics.

[94] Wang S, Dong Y, Gong A (2021). Exosomal circRNAs as novel cancer biomarkers: challenges and opportunities. Int J Biol Sci.

[95] Xu C, Jun E, Okugawa Y (2024). A circulating panel of circRNA biomarkers for the noninvasive and early detection of pancreatic ductal adenocarcinoma. Gastroenterology.

[96] Nikanjam M, Kato S, Kurzrock R (2022). Liquid biopsy: current technology and clinical applications. J Hematol Oncol.

[97] Ma H, Bell KN, Loker RN (2021). qPCR and qRT-PCR analysis: regulatory points to consider when conducting biodistribution and vector shedding studies. Mol Ther Methods Clin Dev.

[98] Gerdes L, Iwobi A, Busch U, Pecoraro S (2016). Optimization of digital droplet polymerase chain reaction for quantification of genetically modified organisms. Biomol Detect Quantif.

[99] Hu T, Ke X, Yu Y (2025). NAPTUNE: nucleic acids and protein biomarkers testing via ultra-sensitive nucleases escalation. Nat Commun.

[100] Wang Z, Gerstein M, Snyder M (2009). RNA-Seq: a revolutionary tool for transcriptomics. Nat Rev Genet.

[101] Nazarov PV, Muller A, Kaoma T (2017). RNA sequencing and transcriptome arrays analyses show opposing results for alternative splicing in patient derived samples. BMC Genomics.

[102] Yang T, Zhang M, Zhang N (2022). Modified Northern blot protocol for easy detection of mRNAs in total RNA using radiolabeled probes. BMC Genomics.

[103] Monné Rodríguez JM, Frisk AL, Kreutzer R (2023). European Society of Toxicologic Pathology (Pathology 2.0 Molecular Pathology Special Interest Group): review of *in situ* hybridization techniques for drug research and development. Toxicol Pathol.

[104] Goytain A, Ng T

[105] Wang S, Qian L, Cao T (2022). Advances in the study of circRNAs in tumor drug resistance. Front Oncol.

[106] Wang X, Wang L, Lin H (2024). Research progress of CTC, ctDNA, and EVs in cancer liquid biopsy. Front Oncol.

[107] Turner N, Huang-Bartlett C, Kalinsky K (2023). Design of SERENA-6, a phase III switching trial of camizestrant in *ESR1*-mutant breast cancer during first-line treatment. Future Oncol.

[108] Tan S, Gou Q, Pu W (2018). Circular RNA F-circEA produced from EML4-ALK fusion gene as a novel liquid biopsy biomarker for non-small cell lung cancer. Cell Res.

[109] Ge L, Sun Y, Shi Y (2022). Plasma circRNA microarray profiling identifies novel circRNA biomarkers for the diagnosis of ovarian cancer. J Ovarian Res.

[110] Borkar S, Markus F, Oetting A (2025). Detection of ESR1 mutations in tissue and liquid biopsy with novel next-generation sequencing and digital droplet PCR assays: insights from multi-center real life data of almost 6000 patients. Cancers.

[111] Kumar MA, Baba SK, Sadida HQ (2024). Extracellular vesicles as tools and targets in therapy for diseases. Signal Transduct Target Ther.

[112] Hama Faraj GS, Hussen BM, Abdullah SR (2024). Advanced approaches of the use of circRNAs as a replacement for cancer therapy. Noncoding RNA Res.

[113] Tao X, Ke X, Xu G (2025). *Mechanisms of circular RNA in drug resistance of lung cancer: therapeutic targets, biomarkers, and future research directions. Discov Oncol.

[114] Kirio K, Patop IL, Anduaga AM (2025). Circular RNAs exhibit exceptional stability in the aging brain and serve as reliable age and experience indicators. Cell Rep.

[115] Rashedi S, Mardani M, Rafati A (2022). Circular RNAs as prognostic and diagnostic biomarkers in renal cell carcinoma. J Clin Lab Anal.

[116] Fosse V, Oldoni E, Bietrix F, PERMIT group (2023). Recommendations for robust and reproducible preclinical research in personalised medicine. BMC Med.

[117] Kuwamoto-Imanishi S, Fujii H (2025). Functions and potential clinical applications of circular RNAs in hepatocellular carcinoma. Hepatoma Res.

[118] Shi H, Zhou Y, Jia E (2022). Comparative analysis of circular RNA enrichment methods. RNA Biol.

[119] Karagianni K, Bibi A, Madé A, EU-CardioRNA COST Action CA17129 (2024). Recommendations for detection, validation, and evaluation of RNA editing events in cardiovascular and neurological/neurodegenerative diseases. Mol Ther Nucleic Acids.

[120] Liu H, Hao W, Yang J, Zhang Y, Wang X, Zhang C (2023). Emerging roles and potential clinical applications of translatable circular RNAs in cancer and other human diseases. Genes Dis.

[121] Antoniou M, Kolamunnage-Dona R, Wason J (2019). Biomarker-guided trials: challenges in practice. Contemp Clin Trials Commun.

[122] Alqahtani S, Alqahtani T, Venkatesan K (2025). Unveiling pharmacogenomics insights into circular RNAs: toward precision medicine in cancer therapy. Biomolecules.

[123] Malviya A, Bhuyan R (2023). The recent advancements in circRNA research: from biogenesis to therapeutic interventions. Pathol Res Pract.

[124] Betz M, Massard V, Gilson P (2023). ESR1 gene mutations and liquid biopsy in ER-positive breast cancers: a small step forward, a giant leap for personalization of endocrine therapy?. Cancers.

[125] Li Q, Geng S, Luo H (2024). Signaling pathways involved in colorectal cancer: pathogenesis and targeted therapy. Signal Transduct Target Ther.

[126] Cui YB, Wang LJ, Xu JH (2024). Recent progress of circRNAs in hematological malignancies. Int J Med Sci.

[127] Li W, Liu JQ, Chen M, Xu J, Zhu D (2022). Circular RNA in cancer development and immune regulation. J Cell Mol Med.

[128] de Gonzalo-Calvo D, Karaduzovic-Hadziabdic K, Dalgaard LT (2024). Machine learning for catalysing the integration of noncoding RNA in research and clinical practice. EBioMedicine.

[129] Pedraz-Valdunciel C, Rosell R (2021). Defining the landscape of circRNAs in non-small cell lung cancer and their potential as liquid biopsy biomarkers: a complete review including current methods. Extracell Vesicles Circ Nucl Acids.

[130] Shi Y, Song R, Wang Z (2021). Potential clinical value of circular RNAs as peripheral biomarkers for the diagnosis and treatment of major depressive disorder. EBioMedicine.

